# How do we cultivate in England? Tillage practices in crop production systems

**DOI:** 10.1111/sum.12241

**Published:** 2015-12-11

**Authors:** T. J. Townsend, S. J. Ramsden, P. Wilson

**Affiliations:** ^1^ Division of Agricultural and Environmental SciencesUniversity of NottinghamSutton Bonington CampusCollege RoadSutton BoningtonLoughboroughLE12 5RDUK

**Keywords:** Agriculture, arable, cultivation, soil management, soil policy, tillage

## Abstract

Reducing tillage intensity offers the possibility of moving towards sustainable intensification objectives. Reduced tillage (RT) practices, where the plough is not used, can provide a number of environmental and financial benefits, particularly for soil erosion control. Based on 2010 harvest year data from the nationally stratified Farm Business Survey and drawing on a sub‐sample of 249 English arable farmers, we estimate that approximately 32% of arable land was established under RT, with 46% of farms using some form of RT. Farms more likely to use some form of RT were larger, located in the East Midlands and South East of England and classified as ‘Cereals’ farms. Application of RT techniques was not determined by the age or education level of the farmer. Individual crops impacted the choice of land preparation, with wheat and oilseed rape being more frequently planted after RT than field beans and root crops, which were almost always planted after ploughing. This result suggests there can be limitations to the applicability of RT. Average tillage depth was only slightly shallower for RT practices than ploughing, suggesting that the predominant RT practices are quite demanding in their energy use. Policy makers seeking to increase sustainable RT uptake will need to address farm‐level capital investment constraints and target policies on farms growing crops, such as wheat and oilseed rape, that are better suited to RT practices.

## Introduction

Lowering tillage intensity is a management practice that potentially can reduce environmental impacts and improve agricultural outputs (i.e. providing ‘sustainable intensification’; Buckwell *et al*., 2014). A number of tillage systems forego the use of the plough (i.e. do not involve soil inversion). These systems vary in the extent to which soil is disturbed, ranging from extensive, in deep reduced tillage (RT^1^), to limited, in shallow RT and minor, in zero‐tillage (ZT) with the latter establishing the crop with only very minimum soil disturbance (Davies & Finney, 2002). These tillage systems are often part of wider agronomic practices that include residue management and, in conservation agriculture, continuous groundcover and diverse crop rotations. The definition of tillage systems varies among practitioners; Table 1 provides an overview summary of these typical tillage/establishment systems. In this paper ‘reduced tillage’ is used to refer to cultivation systems that do not involve soil inversion.

**Table 1:** Description of types of tillage practices. NB tillage definitions in the literature vary widely and may differ from those given in this table

Tillage type	Description
Conventional tillage	Conventional tillage usually relates to ploughing, which involves inversion of the soil with the purpose of loosening the soil and burying weeds and residues from the previous crop. Generally, ploughing is followed by secondary tillage, such as powered or unpowered harrows/discs, although not always such as on lighter soils. [NB Some definitions of conventional tillage include deep noninversion tillage]
Noninversion tillage; reduced cultivation; reduced tillage; minimum tillage	These are tillage practices that do not invert the soil. Some definitions specify maximum cultivation depths (e.g. no greater than 100 mm) and/or a particular percentage cover, usually 30% of crop residues left on the soil surface
Deep reduced tillage	Noninversion tillage to a depth greater than 100 mm/150 mm
Shallow reduced tillage	Noninversion tillage to a depth of less than 100 mm
Strip‐tillage	Strips (covering less than a third of the soil surface) are tilled and the residue moved onto the untilled strips. Seeds are then drilled on the tilled strips
Zero‐tillage/no‐till/direct drilling	This is where the seed is drilled into the stubble of the previous crop with only very minor soil disturbance
Conservation tillage	Reduced tillage combined with at least 30% residue cover, where water erosion predominates, or at least 1120 kg crop residue left on the surface, where wind erosion predominates
Conservation agriculture	Zero‐tillage combined with permanent organic soil cover (either residue or cover crop), and diverse crop rotations
Mixed tillage	A farm system that uses both conventional tillage and reduced tillage. This can take the form of rotational ploughing or strategic tillage
Rotational ploughing	A system where the land is ploughed at specific points in the rotation with other tillage practices used in between
Strategic tillage	A flexible, responsive system where ploughing is used within the rotation in response to specific conditions
Secondary tillage	This term tends to refer to shallower and finer‐scale tillage practices occurring after the main tillage practice

Previous authors have identified environmental benefits of reducing tillage intensity in terms of: reduced soil erosion, pesticide runoff, nitrate leaching and watercourse sedimentation; improved soil quality and smaller greenhouse gas emissions (Fawcett & Towery, 2002; Holland, 2004; Morris *et al*., 2010). Other benefits include reduced fuel costs, improved timeliness of field preparation, less machinery input required and lower machinery expenses through lower wear and tear (Baker *et al*., 2007; SoCo Project Team, 2009). Across a range of studies conducted in northern Europe, labour requirements were 30–40% lower for RT systems relative to ploughing (SoCo Project Team, 2009); fuel use was also much lower, although this was greatly dependent on specific machinery used and the soil conditions. Throughout the literature, there is inconsistency in the impacts of adopting RT practices and this is due to the variation in RT practices used as well as the specific cropping systems, soil types and climate.

Reduced tillage is widely practised in North and South America, with increasing uptake in South Africa, Australia and other semi‐arid areas where the primary driver for uptake is reduction in soil erosion (Holland, 2004). In humid temperate regions, such as Northwest Europe, where soil erosion is less of a problem, the main reason for the use of RT is cost savings (Morris *et al*., 2010). Davies & Finney (2002) reviewed the use of RT in the UK and suggested that using RT practices offered the best opportunity for reducing labour and machinery costs and, therefore, reducing production costs and increasing profit.

Estimates for the UK suggest that one‐third of crops grown on cereal‐growing land were established under RT in the 1970s; however, problems with grass weeds led to a reduction in use to about 10% in 1988 (Davies & Finney, 2002). During that time, straw burning facilitated the use of RT because residues left *in situ* can interfere with seed drilling and result in increased disease and pest incidence (Carter, 1994). In the absence of straw burning, these issues have been previously cited as constraints to the use of RT (Cannell, 1985). The phasing out of straw burning, which culminated in a ban on the practice in 1993 for England and Wales, further encouraged use of the plough. More recently, RT use has been increasing: Defra's 2010 *Farm Practices Survey* found that 44% of arable land in England was under RT (with at least 30% of residue coverage) with 4% of this under ZT (Defra, 2010). Data from the 2010 *Survey on Agricultural Production Methods* (SAPM) show that tillage practices vary throughout Europe (EuroStat, 2013). RT in the UK^2^ was estimated as 39%, which is similar to Germany (41%) and France (36%), yet much greater than the Europe‐wide average of 26%. ZT covered 5% of arable land in the UK whilst Germany and France had 1% and 4%, respectively. Although these national usage figures exist, there is limited data available on specific tillage practices at the farm level. However, interest in adoption of RT may be increasing as it is recognized as an important management practice and the Soil Protection Review, which must be completed by every farmer receiving payments as part of the Common Agricultural Policy in the UK, encourages the use of RT practices (Anon, 2010). In addition, policy makers are seeking to incentivize sustainable intensification practices to achieve greater levels of production from lower input use (e.g. via the UK funded Agri‐Tech programme). RT is recognized as a practice contributing to sustainable intensification.

Although the area under RT appears to be increasing, there are constraints to its wider adoption (Powlson *et al*., 2012). The feasibility of adopting RT depends on soil type and climate (Cannell *et al*., 1978; Carter, 1994; Davies & Finney, 2002; Morris *et al*., 2010). In particular, good drainage and a naturally stable structure are desirable to avoid soil compaction. Calcareous soils with or without large clay contents are more suited to RT (Davies & Finney, 2002) than light sandy soils (Morris *et al*., 2010). Where ZT is practised in the UK, it tends to be on calcareous clay soils, which ‘self‐mulch’ (Powlson *et al*., 2012). It can be assumed that these constraints to adoption are likely to be greater the lower the level of tillage intensity.

Reduced tillage systems in much of Northwest Europe tend to require greater herbicide use to control weeds (Melander *et al*., 2013). Even with additional herbicide use, weed problems can limit the use of RT. In particular, black‐grass (*Alopecurus myosuroides* Huds.) is the major weed of winter cereals and one method of control is mouldboard ploughing (see, e.g. Lutman *et al*., 2013). Due to this, a common approach in the UK is to use RT practices in conjunction with ploughing; Powlson *et al*. (2012) refer to this as *rotational ploughing*, whereby the plough is used every 3 to 4 yrs. There is not, however, a strict definition of *rotational ploughing,* and it is likely that farms range from using ploughing at a fixed point within a set rotation to using ploughing in response to specific conditions (e.g. after outbreaks of disease or pests). In Table 1 these are broadly defined as *rotational ploughing* and *strategic tillage*, respectively, with the term *mixed tillage system* referring to farm systems utilizing both ploughing and RT.

Although there has been considerable work investigating the impact of continuous ZT on cropping systems and environmental impacts relative to yearly ploughing (see Soane *et al*., 2012 for a review), work quantifying changes resulting from other RT systems and mixed tillage systems compared to yearly ploughing is limited and results are often confounded by location and specific cultivation practice outcomes. Dang *et al*. (2015a,b) reviewed the literature on using strategic tillage in ZT systems and found that impacts on cropping systems and environmental impacts were highly variable.

Evidence from field experiments comparing different tillage systems shows a slight reduction in crop yields under RT (Van den Putte *et al*., 2010; Arvidsson *et al*., 2014); however, yield impacts varied with crop type, tillage depth and crop rotation. When individual field experiments are considered, yields can be higher under RT (e.g. Knight, 2004; Verch *et al*., 2009). Yield data for different tillage systems in the UK are, however, limited and moreover typically relate to experiments that were undertaken prior to the ban on straw burning in England and Wales discussed above. As straw burning reduces weed, disease and pest burden for the following crop (Graham *et al*., 1986), these studies are unlikely to reflect current evidence on the impact of RT on crop yields. Alongside this, there have been advances in RT machinery, improving the establishment of crops in RT systems. A potential issue with these studies is that crops are drilled simultaneously on ploughed and RT experimental plots; in commercial contexts RT practices tend to be quicker to implement than ploughing allowing earlier crop establishment, which could provide a yield benefit not captured in these studies.

Given this background, the aim of this paper is to present a more current analysis of the uptake of RT systems on English arable farms, providing definitive context to the tillage depth employed within commercial farm practice, the extent of continuous or rotational RT and yield and crop gross margins associated with different tillage systems. Specifically we test the following hypotheses:

- Area of RT is influenced by location, farm type, farmer age and level of education, and farm size.
- Average depth of main type of tillage is influenced by location, farm type, farmer age and level of education, and farm size.
- Choice of tillage depends on crop type.
- Tillage practices influence production metrics.

## Methodology

Data were collected between February and October 2011 in conjunction with a survey of English arable farms, sampled from the Farm Business Survey (FBS) research programme which samples approximately 3% of the commercial farm business in England. The information was obtained at the same time as that reported by Glithero *et al*. (2013a,b,c) on the supply of biomass for biofuel production. A detailed description of the data collection method is provided in Glithero *et al*. (2013c). Analyses in the papers cited above were based on a sample of 249 farms businesses, drawn from the nationally stratified FBS to form a stratified subsample of commercial farm businesses within farm types of interest. Specifically, this subsample represented approximately 46% of FBS farms within the three main arable farm types (Cereals, General Cropping and Mixed). Within the FBS, farm types were classified on the basis of economic output from the enterprises or group of enterprises. When a farm business derived at least 2/3rd of its output from an individual enterprise or grouping of enterprises (e.g. combinable crops output for the Cereals farm type) the farm business was classified as a particular farm type; in the absence of the 2/3rd output threshold being met from a specific enterprise, farms were classified as Mixed. Additionally farms were stratified across three utilized agricultural area groupings within these farm types (Anon, n.d.). These 249 farms covered approximately 1.5% of the total commercial population of these farm types in England. Corresponding individual farm data for the main crop specific gross margins (GMs, i.e. value of total output less variable costs), yields and variable costs were taken from the FBS data for the 2010/11 financial year; the FBS did not record detailed records of residue use (e.g. straw incorporation versus baling); however, data on residue use in England were presented in Glithero *et al*. (2013c) drawing on the survey detailed above. The economic data were provided by the main FBS account for each of the 249 farm businesses. Here, we focus on the financial records at the level of the total output and variable costs for individual crop enterprise without the data relating to the fixed costs of production. Drawing on the combined data sources of the FBS and the survey for each farm we investigated the current use of RT in England, the depth of tillage practices (given as average depth of the main tillage practice), where RT is used within the crop rotation and how tillage practices relate to key input and output data for individual crops.

The farms were aggregated into groups for analysis based on the following characteristics: farm size (based on the Defra's Standard Labour Requirement levels expressed in terms of full‐time equivalents (FTE) was defined as: Small (<2 FTE), Medium (≥2 FTE and <3) or Large (≥3 FTE); farm type was classified as Cereals, General Cropping or Mixed arable and livestock, representative of the main arable farm types in England); location was identified both in terms of Government Office Region, GOR and European Union, EU, super region, which are, respectively, one of nine and one of three groupings within England); education level of the farmer; farmer age; and ploughing frequency before specific crops (i.e. ‘ploughing always’, ‘ploughing sometimes’ and ‘ploughing never’). The percentage area of RT was determined using area weighting based on crop areas, GOR and farm type to aggregate results to be nationally representative of total area. The ‘area weights’ are derived from calculating the population areas of wheat, barley and oilseed rape, divided by the survey areas for crop, farm type and GOR. This provides area‐weighting values by crop, GOR and farm type. These area weights are then applied to the individual farm results to provide nationally representative aggregate estimates (Glithero *et al*., 2013c).

Data were analysed using GenStat (VSN International Ltd.). RT use was compared among groups, after dividing farms based on whether they did or did not use RT practices, using chi‐square with a 5% significance level. For all other analyses ANOVA was used at the 5% significance level after checking that the test assumptions were met. Variable costs, yields and gross margins were compared among groups varying in the frequency of ploughing for four crops. For comparison of tillage depth among groups, one farm, which had 100% RT, was excluded as it did not provide data on the depth of cultivation (it was possibly 100% direct drilled). The Bonferroni *post hoc* test (set at 10% significance level) was used after the ANOVA to determine significantly different groups.

## Results

### Area of RT

**Figure 1:**
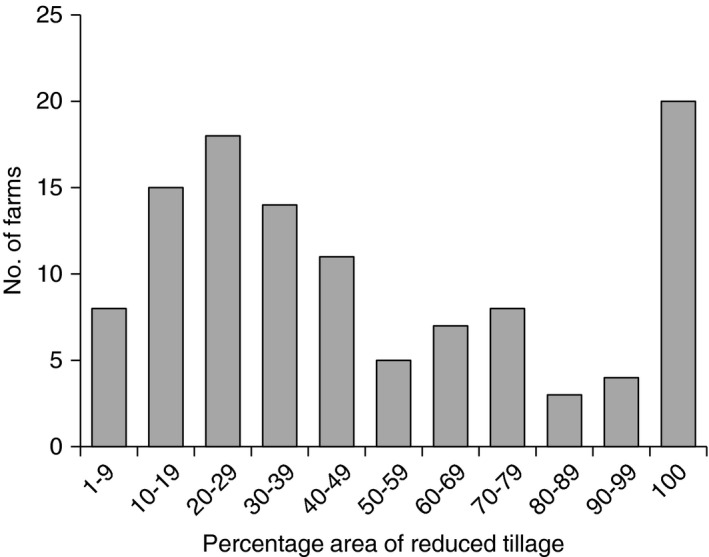
The number of farms with set areas of reduced tillage.

Of the 249 farms surveyed, 113 (46%) used at least some form of RT; an additional 14 farms had used or were likely to use RT within the rotation, suggesting that over half of the farms surveyed used RT at some point. Based upon aggregation weighting, we identified that 32% of the arable land was subject to RT practices. For farms that used RT, the mean area under RT was 50%; however, the amount of RT on individual farms varied greatly (Figure 1). There were eight farms with >10% of the area under RT and 20 farms that did not plough at all.

Of the farm types, Cereal farms were more likely to use at least some RT (*P* < 0.001; data for the following analyses are presented in Table 2); however, where RT is used, farm types do not differ in the percentage area of RT (*P* = 0.548). Large farms were much more likely to use some RT than small farms (*P* < 0.001). For farms that used RT, farm size did not influence the proportion of land under RT (*P* = 0.090).

**Table 2:** Proportion of farms using reduced tillage (RT), the area under RT on these farms and mean depth of tillage for farm groupings. Farm size: groupings based on Defra's Standard Labour Requirement levels; Farm type: farm groupings classified on the basis of the most dominant 2/3rd of economic output (as quantified by UK Defra's Standard Output definitions); GOR: Government Office Region; Farmer age: age of principal farm manager in years old. Statistical analysis: chi‐square (χ^2^) and ANOVA F values and corresponding degrees of freedom (DF), and P values; superscript letters refer to Bonferroni groupings at 10% significance level

		Total number of farms	Number of farms using RT (percentage of farms)	Mean percentage area of RT for farms with RT (standard error)	Mean tillage depth (standard error)
Farm size	Small	65	14 (22%)	62.0 (±8.80)	19.3 (±0.62)
	Medium	119	59 (50%)	44.2 (±3.90)	19.7 (±0.52)
	Large	62	43 (69%)	54.9 (±5.27)	21.5 (±0.69)
	χ^2^ (DF)	–	29.66 (2)	–	–
	F. stat. (DF)	–	–	2.47 (2, 100)	2.68 (2, 245)
	*P* value	–	<0.001	0.09	0.07
Farm type	Cereal	124	78 (63%)	51.8 (±3.73)	19.6^a^ (±0.43)
	General Cropping	57	17 (30%)	42.6 (±6.28)	22.3^b^ (±0.84)
	Mixed	65	21 (32%)	51.4 (±8.02)	19.1^a^ (±0.67)
	χ^2^ (DF)	–	24.96 (2)	–	–
	F. stat. (DF)	–	–	0.61 (2, 110)	6.80 (2, 245)
	*P* value	–	<0.001	0.548	0.001
GOR	North East	15	9 (60%)	25.6 (±6.52)	19.0^a,b,c^ (±1.02)
	North West	16	4 (25%)	36.9 (±12.59)	19.5^a,b,c^ (±1.08)
	Yorkshire & the Humber	29	8 (28%)	27.4 (±6.16)	18.8^a,b^ (±1.13)
	East Midlands	46	28 (61%)	55.1 (±6.09)	20.3^a,b,c^ (±0.80)
	West Midlands	20	8 (40%)	50.4 (±12.79)	21.3^b,c^ (±1.01)
	East of England	63	30 (48%)	49.7 (±5.84)	22.6^c^ (±0.60)
	South East	32	19 (59%)	70.2 (±7.12)	16.9^a^ (±0.98)
	South West	28	7 (25%)	44.3 (±10.42)	18.9^a,b^ (±0.85)
	χ^2^ (DF)	–	19.71 (7)	–	–
	F. stat. (DF)	–	–	2.95 (7, 105)	5.55 (7, 240)
	*P* value	–	0.006	0.007	<0.001
Region	North	60	23 (38%)	28.5^a^ (±4.21)	19.00 (±0.66)
	East	141	79 (56%)	56.7^b^ (±3.70)	20.9 (±0.50)
	West	48	17 (35%)	47.6^a,b^ (±8.13)	19.9 (±0.67)
	χ^2^ (DF)	–	8.93 (2)	–	–
	F. stat. (DF)	–	–	7.22 (2, 110)	2.12 (2, 245)
	*P* value	–	0.011	0.001	0.123
Farmer age	20–39	7	3 (43%)	52.9 (±23.69)	22.4 (±1.45)
	40–49	58	24 (41%)	48.4 (±6.24)	20.0 (±0.68)
	50–59	80	36 (45%)	44.3 (±5.20)	19.8 (±0.51)
	60–69	77	33 (43%)	64.1 (±5.86)	20.0 (±0.65)
	70+	27	17 (63%)	38.3 (±5.88)	21.0 (±1.40)
	χ^2^ (DF)	–	3.96	–	–
	F. stat. (DF)	–	–	2.61 (4, 108)	0.66 (4, 243)
	*P* value	–	0.411	0.039	0.692
Education	No formal qualifications	68	31 (46%)	55.0 (±5.89)	19.7 (±0.70)
	General Certificate of Secondary Education, A level or equivalent	35	15 (43%)	61.1 (±8.93)	20.4 (±0.91)
	College/National Diploma/certificate	105	47 (45%)	43.9 (±4.17)	20.1 (±0.50)
	Higher education degree	41	20 (49%)	49.8 (±7.90)	20.1 (±0.98)
	χ^2^ (DF)	–	0.30 (3)	–	–
	F. stat. (DF)	–	–	1.45 (3, 109)	0.13 (3, 245)
	*P* value	–	0.960	0.232	0.944

The number of farms using RT varied with GOR (*P* = 0.033) with the East Midlands and South East more likely to have some RT, whereas the North West, Yorkshire & The Humber and South West were more likely to have none. For farms where RT was used, the North East and Yorkshire & the Humber had significantly smaller areas of RT than the South East (*P* = 0.007). When these GOR regions were aggregated to EU super regions (East, West and North), farms in the East were more likely to have some RT than farms in the North and the West (*P* = 0.011). Where at least some RT is used, farms in the East had significantly greater areas of RT than those in the North (*P* = 0.001).

Whether farmers are using RT or not does not depend on age (*P* = 0.411) or education (*P* = 0.960). For farms that use some RT, the area of RT did not vary with education level (*P* = 0.232) but did with age group (*P* = 0.039), with farmers in the 60–69 age group having the largest percentage area of RT and farmers in the 70+ age group having the smallest percentage area of RT.

### Depth of tillage

**Figure 2:**
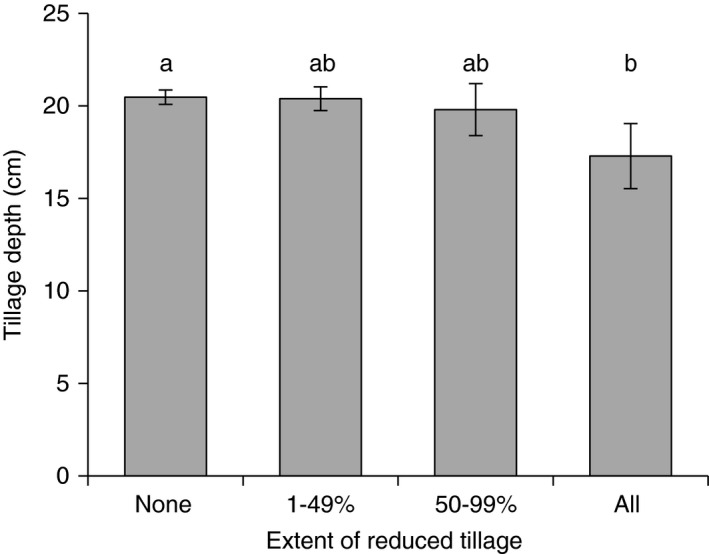
Average depth of main tillage practice for farms with differing areas of reduced tillage. Mean values: None = 20.47 cm; 1–49% = 20.39 cm; 50–99% = 19.80 cm; All = 17.29 cm. ANOVA:* F*_(3,__244)_ = 2.06; *P* = 0.105. Letters represent Bonferroni test (at 10% sig. level). Error bars show standard error.

Average tillage depth of the main form of tillage for all surveyed farms was 20 cm. Shallower tillage depth tended to be associated with farms with all RT (*P* = 0.105; Figure 2) although their mean depth was only 3 cm shallower than the mean value when only ploughing was used. There was considerable variation in tillage depths, in particular for farms using all RT; for example, four respondents tilled to a depth of 0–10 cm, seven respondents tilled to a depth from 10 to 20 cm while eight respondents tilled to a depth over 20 cm. As there were only a small number of farms that had an average tillage depth of less than 10 cm it was not possible to identify specific characteristics relating to these farms.

Depth of tillage varied with farm type, being significantly deeper on General Cropping farms compared to Cereal and Mixed farm types (*P* < 0.001). There was a strong trend for deeper tillage on Large farms (*P* = 0.070) but the difference in average tillage depths between farm size groups was very small. Tillage depth was significantly greater in the East of England than in the South East (*P* < 0.001); however, when aggregated into EU super regions there was no significant difference (*P* = 0.193). There was no difference in tillage depth with level of education (*P* = 0.944) or age (*P* = 0.624).

### Reduced tillage in the rotation

**Figure 3:**
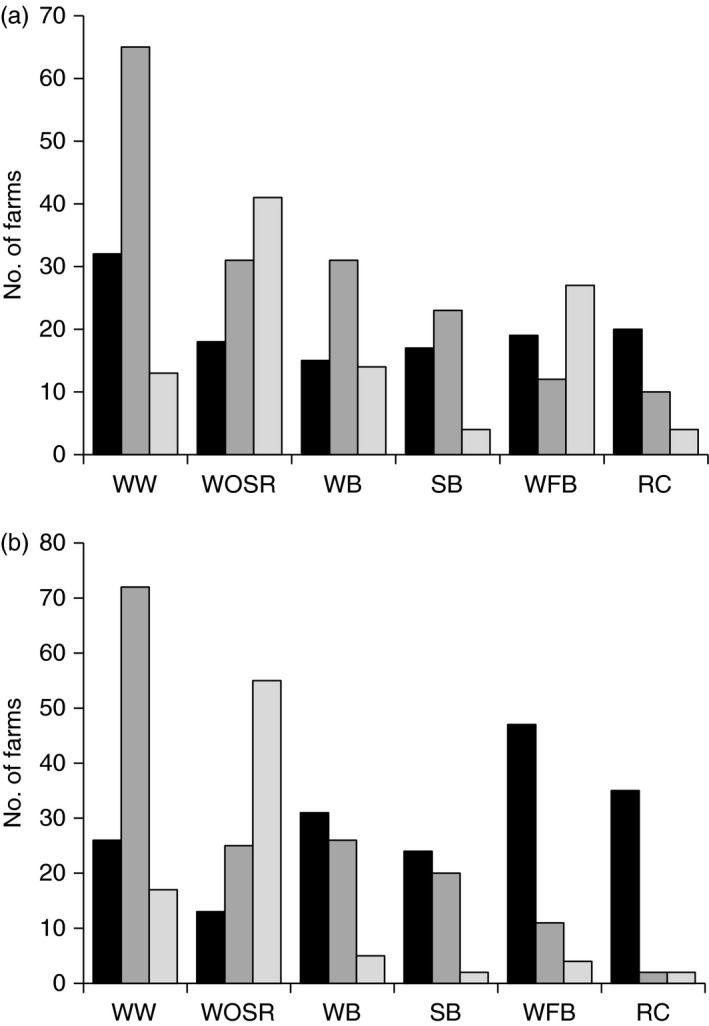
Frequency of ploughing before (a) and after (b) different crops. Always plough (black bars); sometimes plough (dark grey bars); and never plough (light grey bars). WW (winter wheat); WOSR (winter oilseed rape); WB (winter barley); SB (spring barley); WFB (winter field beans and peas); RC (root crops).

Analysing data only for farms that used *some* RT, the frequency of ploughing (i.e. whether they *always plough* [AP], *sometimes plough* [SP] or *never plough* [NP]) for different crops was considered both before the crop (Figure 3a) and after the crop (Figure 3b). Decisions about whether to plough before a crop will in part depend on the preceding crop; however, without knowledge of the specific rotations used on these farms it was not possible to explore this.

Reduced tillage is often used with winter wheat (WW) and winter oilseed rape (WOSR), less so with winter barley (WB), spring barley (SB) and winter field beans (WFB), and very little with root crops (i.e. potatoes and sugar beet; RC). As rotations are built around WW and WOSR, for farms with these crops it also tends to be used after these crops. For before and after WW, the majority of respondents recorded they sometimes plough; this was also a common response for WOSR and barley, demonstrating flexibility in the cultivation approach. Although WFB and RC are almost always planted after ploughing, WFB tended to be followed by RT while RC are almost always followed by ploughing.

### Yields, gross margins and input costs

Yield and gross margin data for each crop were tested to assess the effect of frequency of ploughing (Tables 3, 4, 5, 6). Farms were excluded that did not grow the specific crop or where gross margin data were not available; hence, the number of farm observation differs from those presented above. For farms that *sometimes plough* before a particular crop, we do not know if they ploughed or used RT; therefore, we take this as an indication that they adopt a ‘flexible’ management approach where tillage practice is varied to provide the most benefit.

**Table 3:** Yield, gross margin and variable cost data for winter wheat with different ploughing frequencies. Frequency of ploughing before winter wheat: *always plough* (AP); *sometimes plough* (SP); *never plough* (NP). *F* and *P* values refer to ANOVA. Superscript letters differentiate groups based on the Bonferroni *post hoc* test at the 10% sig. level. Degrees of freedom: 2, 197. Other variable costs include grain drying fuel and other miscellaneous costs. Standard errors in parenthesis

	Ploughing frequency			*F* value	*P* value
	AP	SP	NP		
No. of farms	112	62	26	–	–
Gross margins (£/ha)	953 (±26.8)	945 (±38.6)	907 (±58.5)	0.27	0.766
Yields (t/ha)	7.81 (±0.17)	8.35 (±0.20)	8.29 (±0.27)	2.45	0.089
Spray costs (£/ha)	136^a^ (±4.6)	154^b^ (±5.9)	163^b^ (±6.4)	5.21	0.006
Fertilizer costs (£/ha)	160 (±6.2)	160 (±6.5)	161 (±10.7)	0.16	0.856
Seed costs (£/ha)	56 (±1.9)	59 (±2.9)	51 (±2.9)	1.40	0.248
Other variable costs (£/ha)	28	24	20	–	–

**Table 4:** Yield, gross margin and variable cost data for winter oilseed rape with different ploughing frequencies. Frequency of ploughing before winter oilseed rape: always plough (AP); sometimes plough (SP); never plough (NP). *F* and *P* values refer to ANOVA. Degrees of freedom: 2, 72. Other variable costs include grain drying fuel and other miscellaneous costs. Standard errors in parenthesis

	Ploughing frequency			*F* value	*P* value
	AP	SP	NP		
No. of farms	40	22	13	–	–
Gross margins (£/ha)	815 (±40.3)	739 (±117.9)	785 (±80.0)	0.27	0.764
Yields (t/ha)	3.76 (±0.11)	3.60 (±0.29)	3.61 (±0.17)	0.38	0.686
Spray costs (£/ha)	138 (±7.5)	143 (±22.1)	153 (±8.5)	0.55	0.581
Fertilizer costs (£/ha)	163 (±7.9)	175 (±16.1)	159 (±9.6)	0.43	0.650
Seed costs (£/ha)	51 (±2.6)	49 (±6.3)	50 (±5.4)	0.04	0.965
Other variable costs (£/ha)	23	24	14	–	–

**Table 5:** Yield, gross margin and variable costs data for winter barley with different ploughing frequencies. Frequency of ploughing before winter barley: *always plough* (AP); *sometimes plough* (SP); *never plough* (NP). *F* and *P* values refer to ANOVA. Superscript letters differentiate groups based on the Bonferroni *post hoc* test at the 10% significance level. Degrees of freedom: 2, 102. Other variable costs include grain drying fuel and other miscellaneous costs. Standard errors in parenthesis

	Ploughing frequency			*F* value	*P* value
	AP	SP	NP		
No. of farms	77	20	8	–	–
Gross margins (£/ha)	731^b^ (±30.6)	733^b^ (±65.6)	412^a^ (±79.3)	5.15	0.007
Yields (t/ha)	6.77^a^ (±0.15)	7.70^b^ (±0.31)	6.12^a^ (±0.49)	5.08	0.008
Spray costs (£/ha)	107 (±3.9)	126 (±10.3)	96 (±11.5)	2.81	0.065
Fertilizer costs (£/ha)	137 (±4.6)	146 (±9.7)	144 (±26.1)	0.35	0.703
Seed costs (£/ha)	54^a^ (±2.0)	70^b^ (±6.0)	65^a,b^ (±9.3)	5.09	0.008
Other variable costs (£/ha)	21	24	99	–	–

**Table 6:** Yield, gross margin and variable costs data for spring barley with different ploughing frequencies. Frequency of ploughing before spring barley: *always plough* (AP); *sometimes plough* (SP); *never plough* (NP). *F* and *P* values refer to ANOVA. Degrees of freedom: 2, 64. Other variable costs include grain drying fuel and other miscellaneous costs. Standard errors in parenthesis

	Ploughing frequency			*F* value	*P* value
	AP	SP	NP		
No. of farms	67	8	4	–	–
Gross margins (£/ha)	577 (±31.2)	698 (±160.9)	516 (±99.1)	0.87	0.424
Yields (t/ha)	5.09 (±0.17)	5.40 (±0.61)	5.12 (±0.56)	0.19	0.825
Spray costs (£/ha)	75 (±4.9)	99 (±13.8)	60 (±9.0)	2.11	0.129
Fertilizer costs (£/ha)	101 (±7.0)	98 (±13.6)	116 (±25.5.)	0.18	0.832
Seed costs (£/ha)	50 (±2.5)	66 (±5.3)	63 (±15.6)	2.91	0.062
Other variable costs (£/ha)	18	30	28	–	–

For WW, yield was lowest for AP farms (*P* = 0.089; Table 3). Spray costs were significantly greater for NP farms than AP farms (*P *= 0.006). GMs, fertilizer costs and seed costs do not vary significantly with the frequency of ploughing. There were no significant differences among the three ploughing frequency groups regarding input and output data for WOSR (Table 4). For WB, yields were significantly greater on SP farms (*P *= 0.008) while GMs were significantly lower for NP farms (*P *= 0.007; Table 5). Spray costs for NP farms were not significantly different from AP farms (*P *= 0.065). Seed costs were significantly greater for SP farms (*P *= 0.008). Fertilizer costs did not significantly differ with ploughing frequency. For SB yield, input costs and GM data did not vary significantly with frequency of ploughing (*P *= 0.062; Table 6).

## Discussion

### Use of RT

Our estimate of 32% of land under RT is less than a previous contemporaneous UK estimate of 44% (Defra, 2010). Within Defra's (2010) Farm Practices Survey it is possible that a greater proportion of respondents with an interest in RT may have completed questions about tillage techniques and returned the survey in comparison with farmers with no interest in RT techniques. However, it should be noted that the sample derived for this current study was based upon the stratified sample of the Farm Business Survey for England; this approach reduces potential bias that may occur from postal‐based surveys whereby respondents with an interest in the subject are more likely to respond (Pennings *et al*., 2002).

The current limited data available on RT practices throughout Europe appear to only capture the average aggregate RT use per country rather than data at the individual farm level. From our data, a small number of farms did not plough but the vast majority of farms using RT practices also ploughed (i.e. mixed tillage systems) largely rejecting the concept of a dichotomy of either using *all* RT or *all* ploughing. Typically mixed tillage occurs either at specific points within the rotation (rotational ploughing) or in response to specific conditions (strategic tillage), with rotational ploughing being the more frequently observed.

With respect to tillage practices and individual crops, for WW, the majority of farmers ‘sometimes plough’ prior to crop establishment; the major break crop, WOSR, which is less prone to weeds, can be established by broadcasting seed directly into the stubble from the previous crop (e.g. using an *autocast* system). RC and WFB were almost always ploughed beforehand. Van den Putte *et al*. (2010) found that RC yields were not significantly lower under RT; however, Lahmar (2010) notes that RCs are harder to manage under RT systems. WFB can be established via broadcasting and then incorporating seed directly with a plough, which is a quick method of establishment and suited to the wet soil conditions usually associated with later sowing. However, there is now growing interest in RT drilling of WFB (PGRO, 2015).

Our results indicate that tillage depth for RT, when it was the main tillage practice, was not significantly different from ploughing, contrasting with previous definitions of RT relating to less than 100 mm (Ingram, 2010) or 150 mm (Powlson *et al*., 2012). Hence, even in the absence of soil inversion, substantial soil disturbance still occurs with RT. Cultivation depth may in part relate to the need to bury crop residues or weeds; indeed, Davies & Finney (2002) suggested that shallow RT was unlikely to become commonly used because it is associated with higher risk of yield penalties resulting from an increased grass weed burden. Van den Putte *et al*. (2010) also report that shallow RT depth can result in higher yield penalties.

Our results indicate that RT is most common on larger farms, supporting the findings of the SAPM. Large RT trains require large powered tractors (e.g. 310–400 horsepower; ABC, 2014), more easily financed on larger farms. Moreover, most of the farms recording RT use also undertook some ploughing; it is more feasible for larger farms to hold two types of cultivation equipment. RT may also be more suitable for larger farms due to their ability to cope with the risk burden associated with RT (Lahmar, 2010). It is likely that larger farms also have greater timeliness issues (Melander *et al*., 2013) giving them greater incentive to switch to faster crop establishment methods. Mixed farm types recorded lower use of RT, arguably resulting from the smaller non‐RC areas, reducing the incentive to invest in larger RT machinery and tractors. A greater area of RT and a shallower depth of tillage was recorded in the South East, consistent with the greater number of Cereal farms relative to General Cropping farms and the observation of Cannell *et al*. (1978), which was based on broad soil characteristics, that large parts of the South East were suitable for ZT.

### Tillage effects on yields, costs and the environment

The widespread use of RT identified from this study suggests that farmers are finding benefits from its use. The survey considered the impact of RT on variable costs and yields but based on the limited data, it is not possible to directly determine how beneficial RT practices are after considering fixed costs and dynamic effects. For example, while fuel costs are recorded at the farm level in the FBS, it is not possible to robustly compare fuel use between farms due to the variable use of contract services, which typically include the cost of fuel, versus own machinery use where fuel costs would be recorded separately. However, given these constraints, our results provide informative estimates at the GM level; specifically, GMs only significantly differed with frequency of ploughing for WB. Using RT may also allow other economic benefits; reducing the time required for field preparation could allow farmers to share tillage equipment, providing cost savings, or to undertake contracting work on other farms.

The literature suggests yields tend to be smaller under RT. However, our data do not support the view that for WW there is a yield penalty associated with the use of RT, although WB yields were smaller under RT. This latter result was not due to an interaction between RT usage rate and differences in crop yields among regions. Crop protection costs would be expected to be greater for RT systems because of the need for more of the weed control to depend on herbicides. WW had higher spray costs where RT was used; however, because crop protection cost data were not disaggregated into individual crop protection products, it was not possible to determine which sprays account for this increased cost. In Sweden, WW yields for RT tend to be much smaller when following another cereal crop (Arvidsson *et al*., 2014). Rieger *et al*. (2008) reported that in Switzerland the yield reduction resulted from diseases carried in crop residues, which would suggest that increased crop protection is required when WW follows another cereal. Interestingly, spray costs were not higher for ‘never plough’ before WB and SB; however, it is not possible to confirm whether this reflects a difference in behaviour among farmers (e.g. attitudes to the environment) or whether those that never plough have different rotations that reduce the need for crop protection products used on barley. Contrary to common assumptions, RT systems can be viable with low herbicide usage although this requires careful management, as seen with ZT experiments in South Portugal (Barros *et al*., 2007). Although spray costs were less for RT, seed costs tended to be greater, possibly reflecting a need for greater seed treatment or seed rate to combat weeds. Due to the modest sample size, it is not possible to attribute the extent of these cost differences to tillage practices. For the majority of the output metrics, there were no significant differences among regions; hence, it is unlikely that differences in RT practices were more frequently observed in regions with higher yields. Due to the constraints imposed by sample size, it was also not possible to separate farms based on the average depth of the main tillage type, so the effects of specific RT practices could not be identified.

Based on current RT practices, environmental benefits achieved with RT may not match those indicated in the literature. Morris *et al*. (2010) suggest that, depending on the equipment used, fuel requirements for some deep RT practices can be similar to those of ploughing, limiting greenhouse gas emission reductions. Without fuel‐use data in our current study, it is not possible to assess this. Davies & Finney (2002) question whether the environmental benefits associated with RT seen in field experiments occur at the farm level; field experiments are unlikely to represent mixed tillage systems as observed in this study. Powlson *et al*. (2012) suggest increased soil carbon benefits from RT would be lost under rotational ploughing; however, it is unclear whether other environmental benefits from RT are achieved within the short term, within mixed tillage systems. It is likely that some RT tillage systems, such as ZT, are better suited to continuous use. In general, during the first several years after adoption of ZT crop yields are initially lower relative to ploughing but increase after about 3 yrs of use. Although this could in part be due to time taken by the farmer to learn this new technique, one important factor is that it takes several years for the soil structure to improve suggesting that disruption to soil structure through rotational ploughing would prevent ZT from achieving yield parity with ploughing (Soane *et al*., 2012).

Farmers are encouraged to utilize tillage practices that minimize soil erosion; while the Soil Protection Review encourages RT, the EU's cross‐compliance regulations encourage ploughing to avoid soil erosion caused by soil compaction (Defra, 2014). One aspect of RT that helps reduce soil erosion is maintenance of crop residue on the soil surface; however, the tillage depths recorded in this study suggest that little crop residue is left on the surface, which may arguably limit the soil erosion benefits of RT. Although the wider survey, from which these data were drawn, did collect information on straw use, due to the limited sample size it is not possible to compare tillage practices and straw use. As problems with residues in RT systems have been previously identified as less pronounced where straw is baled (Cannell, 1985), this might support the view that straw baling is more common on RT farms. However, one aim of RT practices is to increase soil organic matter, yet residue removal can negatively impact on soil organic matter levels (Blanco‐Canqui & Lal, 2009). Moreover, contemporary residue management and RT techniques differ substantially from those of the 1980s. In Defra's (2010) Farm Practices Survey, RT is defined as having at least 30% crop residue cover but it is unclear whether this level of residue cover can be achieved with deep RT.^3^ Considering the mean tillage depths recorded in our survey, it may be that the 44% of land under RT with 30% residue cover given in Defra (2010) is unrealistic.

### Increasing the adoption of RT

There are arguments for encouraging greater use of RT practices – wider adoption would address some of the challenges raised under the banner of ‘sustainable intensification’ and the drive for greater productivity at lower environmental cost (Garnett *et al*., 2013) while meeting the demands of a growing, more affluent global population. Climate change – and hence greenhouse gas mitigation – and soil erosion (Defra, 2009) are two areas where RT can play a part and both increasing the use of, and maximizing the environmental benefits from RT represent important interventions that can drive improvements in sustainable intensification. However, as we have seen, RT covers a wide range of practices and is suited to different types of farm situation (for example, the level of grass weed burden) and thus will not be adopted in all situations.

There are thus limits to the extent that RT can be applied in the UK; although Cannell *et al*. (1978) suggested approximately 80% of arable land could be used for ZT, Davies & Finney (2002) concluded that for successful RT, systems must be suited to specific soil types, site, scale and management factors, whereas ploughing is more universally applicable. Although our survey did not consider soil type and climate, the differences observed among regions and farm types support the argument that site‐specific factors influence the applicability of RT. The corollary to this is that site‐specific factors are likely to also limit the extent to which RT can be expanded.

Other factors may constrain further expansion of RT. The biggest concern regarding RT practices is reduced yields (Jones *et al*., 2006); farmers may also be reluctant to use RT due to the tradition of using the plough and concerns that, because RT practices tend to produce fields of less ‘tidy’ appearance, that their land will be perceived by neighbouring farms as being poorly managed – this is probably increasingly the case in the age of ‘Google Earth’. There is also limited assistance for farmers wishing to switch to RT practices; The Soil Management Initiative Ltd (SMI), the UK branch of the European Conservation Agriculture Federation, was established to provide information on RT to farmers (e.g. SMI, 2005). But it is no longer available and there appears to be no organization providing this support.

### Future research and industry recommendations

Our results present a current picture of tillage practice on commercial arable farms in England. Whereas previous research has focused on economic data for single farms, this study considered financial data across a number of farms to provide a better understanding of the variability among farms that utilize RT. While this did allow a better insight into financial impact of RT, it did necessitate a compromise in the comprehensiveness of data collection (e.g. no fuel‐use data for individual crop enterprises). Future approaches to this type of research should explore the rotational and tillage practice effects in combination; for field‐experimental observations, this will require longer‐term studies to be undertaken.

Although providing information on RT use with different crops, the survey did not identify specific RT practices used or the adaptations farmers have made to incorporate RT into their farming systems. Tillage depth measurements were also limited to the average depth of the main tillage practice. Consideration of the specific characteristics of the farms, in particular soil type, would be required to better understand how farm‐specific factors influence the outcomes of using RT. Future survey work should seek to determine the reasons why farmers use RT in order to test the hypothesis that the benefits of RT as established in the literature correlate with commercial practice and observations.

Further work is also needed to quantify short and longer‐term environmental benefits to determine whether deep RT is environmentally beneficial. One potential barrier to future increases in RT is increased weed problems resulting from reduced effectiveness of available herbicides and stricter pesticide legislation limiting crop protection product availability (Melander *et al*., 2013). It is likely that broader changes to farm systems are required to maximize the potential of RT, for example by incorporating aspects of conservation agriculture, such as longer rotations.

Information on ‘best practice’ is limited and providing this information could assist farmers in adopting RT practices. Future research should explicitly generate applied, practical outcomes to facilitate further uptake of RT systems that maximize benefits to farmers and the environment.

## Conclusions

There is widespread use of reduced tillage practices in England, although its use varied with farm size and type, region and specific crops grown. RT tended to be used only on part of the land demonstrating that many farmers practice a rotational ploughing system. The depth of soil disturbance suggests that the RT practices used in England are quite energy demanding, so the benefits of using RT are limited. Where RT is associated with genuine production and environmental benefits, policy makers seeking to achieve sustainable intensification objectives may choose to incentivize the use of RT practices. Our findings indicate that these incentives should address farm‐level constraints such as the capital investment required to hold equipment for sustainable RT cultivation methods; recent (2015) changes in UK investment policy for farms give farmers an incentive to increase investment in new technology by raising the annual investment allowance but do not tie this to particular sustainable practices. Moreover, targeting increased uptake of RT in areas where RT use is already more prevalent, due to climatic or land‐based factors, will facilitate policy success. Finally, in incentivizing RT uptake, policy makers need to be aware of the substantial soil disturbance frequently observed with on‐farm RT use, which provides a potential limit to any environmental benefit that might be anticipated to result from increasing the use of RT in contemporary commercial practice.

## Notes

1 Abbreviations: always plough (AP); Farm Business Survey (FBS); gross margin (GM); never plough (NP); reduced tillage (RT); root crops (RC); sometimes plough (SP); spring barley (SB); survey on agricultural production methods (SAPM); winter barley (WB); winter field beans (WFB); winter oilseed rape (WOSR); winter wheat (WW); zero‐tillage (ZT).

2 Complicating comparisons between information sources is that some statistics are given for the UK as a whole whilst others refer to individual countries within the UK. The data from the survey presented in this paper refer to England.

3 In asking about arable land cultivation, the Farm Practices Survey did not provide an option for RT with less than 30% residue cover.
